# The Impact of Biotechnologically Produced Lactobionic Acid on Laying Hens’ Productivity and Egg Quality during Early Laying Period

**DOI:** 10.3390/ani14202966

**Published:** 2024-10-14

**Authors:** Jelena Zagorska, Diana Ruska, Vitalijs Radenkovs, Karina Juhnevica-Radenkova, Tatjana Kince, Ruta Galoburda, Ilze Gramatina

**Affiliations:** 1 Food Institute, Faculty of Agriculture and Food Technology, Latvia University of Life Sciences and Technologies, LV-3004 Jelgava, Latvia; jelena.zagorska@lbtu.lv (J.Z.); tatjana.kince@lbtu.lv (T.K.); ilze.gramatina@lbtu.lv (I.G.); 2 Institute of Animal Sciences, Faculty of Agriculture and Food Technology, Latvia University of Life Sciences and Technologies, LV-3001 Jelgava, Latvia; diana.ruska@lbtu.lv; 3 Processing and Biochemistry Department, Institute of Horticulture, LV-3701 Dobele, Latvia; karina.juhnevica-radenkova@lbtu.lv; 4 Research Laboratory of Biotechnology, Division of Smart Technologies, Latvia University of Life Sciences and Technologies, LV-3004 Jelgava, Latvia

**Keywords:** amino acids, egg quality, fatty acids, lactobionic acid, laying hens

## Abstract

**Simple Summary:**

Lactobionic acid (Lba) has been found to have a range of health-promoting benefits and practical applications across various fields, including agriculture. However, the impact of Lba on the chemical composition and nutritional quality of eggs from early laying hens has been largely overlooked. The incorporation of Lba into the diet has been observed to elevate the content of monounsaturated fatty acids (MUFAs), specifically palmitoleic and vaccenic acids. In summary, as a functional product, Lba has the potential to improve the productivity of laying hens and enhance the nutritional value of eggs during the early laying period.

**Abstract:**

Lactobionic acid (Lba), an oligosaccharide aldonic acid, has demonstrated various health-promoting benefits and applications in diverse areas. Lba has been recognized for its multifunctional properties, such as metal ion chelation and calcium sequestration. This study aimed to evaluate the effects of supplementing the diet of early-laying hens with Lba (EXP group) on their performance and the physical–chemical properties, and nutritional quality of eggs. The 12-week study involved 700 Sonja breed hens per group, with the EXP group’s diet enriched with 2% of biotechnologically produced Lba, while the control group (CON) received no Lba supplementation. Lba supplementation influenced both the hen’s performance and egg quality, particularly in terms of egg production and fatty acid accumulation. Performance in the EXP group was significantly improved (*p* < 0.05), showing a 4.6–8.9% increase compared to the CON group at all experiment stages. Lba also promoted an increase in monounsaturated fatty acid (MUFA) content, particularly palmitoleic and vaccenic acids. Overall, Lba supplementation enhanced both the productivity of laying hens and the nutritional value of eggs during the early laying period.

## 1. Introduction

Eggs can provide culinary variety while being an economical source of nutrients such as fatty acids (FAs), proteins, and fat-soluble vitamins A, E, and D [1,2]. The internal quality of eggs from laying hens is influenced by various factors, including poultry breed, feed composition, season, and housing system [3]. The edible part of an egg is composed of egg yolk and egg white. The egg yolk mainly comprises phosphoproteins and lipids (fats, phospholipids, steroids, and sterols) [4]. The profile and content of FAs in egg yolk, which are part of triglycerides and phospholipids, strongly depend on the hen’s diet [5]. Until recently, studies have shown that the inclusion of ingredients rich in monounsaturated fatty acids (MUFAs) in the diet of laying hens positively affects the profile of egg yolk FAs [6]. However, Lin et al. [7] reported that the enrichment of the basic diet of laying hens with conjugated linoleic acids (CLAs) and other fats could lead to hyperlipidemia, which is a risk factor for fatty liver disease development, also causing a decrease in egg production and the sudden death of birds. Along with health-promoting FAs, the eggs are also packed with proteins, of which up to 67% are located in egg whites, and they benefit the human organism with all nine essential amino acids (EAAs) [4]. The composition and profile of amino acids (AAs) in eggs strongly depend on poultry breed, age, diet, and housing conditions [8]. Methionine and cysteine, the total sulfur-containing AAs, are the primary limiting AAs in practical diets because of their narrow availability in protein sources of plant origin [9]. Therefore, it has been proposed that supplementing the diet of laying hens with AAs can be a successful strategy for enhancing birds’ performances and the quality traits of eggs such as weight, eggshell strength, shell thickness, and albumen ratio [10].

Lactobionic acid (Lba) has been shown to have a range of health benefits and applications in food processing (food additives) [11], cosmetic production (active compounds) [12], and pharmacy [13]. It has been proven that biotechnologically obtained Lba additive positively affects the overall health of laying hens and the quality traits of eggs [14]. The authors indicated the ability of Lba additive to improve egg production performances, eggshell quality, and feed efficiency. However, a detailed analysis of FAs and AAs, which are also important indicators of egg quality, has not been performed. Recent research has investigated the potential benefits of Lba as a feed supplement for cows and pigs [15,16], documenting the positive effect of Lba on the content of AAs, EAAs in particular, in pork meat and milk. The increase in the content of AAs can be associated with promoting AAs synthesis by cells, as it was reported for extracellular matrix component production by ascorbic acid [17]. Additionally, Lba may have potential as an antimicrobial agent [18], reducing the risk of bacterial infections in laying hens and decreasing antibiotic resistance among poultry. Lba is considered a prebiotic, a valuable substrate for the friendly gut bacteria to compete appropriately with other less desirable bacteria and pathogens, thus promoting optimal gut health in poultry [19]. As a mineral absorption enhancer, acidifier, antioxidant, antibacterial, and prebiotic, Lba has great potential in feed production [20,21]. It is speculated that the Lba can help the laying hens’ body to absorb calcium from the feed and supplements. This phenomenon can be reinforced by organic acids’ ability to bind to calcium ions in feed and help bring them to target organs, making better assimilation of them and reducing their excretion from the body. The solubility of this form of calcium is sixty-five times higher than that of other forms of calcium, such as citrate, which is considered one of the most bioavailable forms [18].

As seen in prior research, Lba has been demonstrated to have a good impact on laying hens’ performance; however, information regarding its influence on the chemical composition and nutritional quality of eggs has been generally underestimated. The abundance of Lba in the whey fraction obtained by taking advantage of optimized fed-batch fermentation conditions developed by the group of Latvia University of Life Sciences and Technologies (LBTU) applying *Pseudomonas taetrolens* DSM 21104 [22] allows putting forward the aim of the study to establish the effect of supplementing the basic diet of early laying hens with Lba (EXP) on their performance, physical–chemical properties, and nutritional quality of the eggs. It was hypothesized that Lba as a functional product could enhance laying hens’ productivity and egg quality during the early laying period. 

## 2. Materials and Methods

### 2.1. Experimental Design

The study was conducted on a farm located in the Vidzeme region of Latvia during the autumn–winter season, 12 weeks in total. Laying hens were divided into control (CON) and experimental (EXP) groups. Each group comprised 700 ± 10 Sonja breed laying hens, delivered at 16 weeks old to the farm. After delivery, each group had ten weeks of acclimatization before starting a trial. Housing conditions for both groups were similar. Laying hens were kept in a cage-free system with an outdoor free walking area in summertime. Lighting conditions in the barn were constant for 16.5 h a day. The basic diet for both groups was prepared on the farm. The feed amount per laying hen was estimated at 125 g per day and fed *ad libitum*. The ingredients and nutritional composition of the diets used in this study are presented in Table 1. A total of 60,000 eggs were produced and collected for the evaluation of the egg production performance per group per experiment.

The physical–chemical features of biotechnologically produced Lba-rich whey used in the experiment as a supplement to the basic diet of laying hens are depicted in Table 2. 

The diet of the EXP group was enriched with 2% Lba liquid fraction by adding it to drinking water. Drinking water was available *ad libitum*. The intake of Lba was controlled by replenishing the container with new water with well-dissolved Lba as the water ran out. A feeding experiment was conducted from weeks 27 to 38 of laying hens age, divided into three stages. During stage 1 from weeks 27–30, stage 2, weeks 31–34, and stage 3, weeks 35–38, the number of eggs laid was recorded daily and sorted by grades (S: less than 53 g, M: 53–62 g, L: 63–72 g, XL: more than 72 g, and cracked) according to Commission regulation (EC) No 589/2008 [23]. The egg production performance (%) was calculated from the total number of eggs laid per day within each stage divided by the total number of hens. Egg samples were obtained three times at the end of each stage to analyze fatty acid and amino acid profiles. At the birds’ age, weeks 30, 34, and 38, ten eggs from each group were randomly collected and delivered to the Latvia University of Life Sciences and Technologies laboratory to evaluate their quality traits and chemical composition.

### 2.2. Chemicals, Standards, and Reagents

A mixture of C_4_–C_24_ fatty acid methyl esters (FAMEs), amino acids (AAs) (purity ≥ 99.0%), methanol (MeOH), acetonitrile (MeCN), *n*-hexane, phenol (C_6_H_5_OH, purity > 99%), and formic acid (HCOOH, purity ≥ 98.0%) of LC-MS grade were purchased from Sigma-Aldrich Chemie Ltd. (St. Louis, MO, USA). Ammonium hydroxide solution (25% *v*/*v*) and diethyl ether (purity ≥ 99.5%) were acquired from Chempur (Piekary Śląskie, Silesia, Poland). Hydrochloric acid (37% *v*/*v*) was purchased from VWR™ International, GmbH (Darmstadt, Germany). Sodium hydroxide, potassium hydroxide, phenolphthalein, and 0.5 M trimethylphenylammonium hydroxide solution (TMPAH) in methanol for GC derivatization were of reagent grade and were obtained from Sigma-Aldrich Chemie Ltd. (St. Louis, MO, USA).

### 2.3. Sample Preparation and Analysis of Fatty Acids by GC-MS

The lipid fraction was prepared using the method outlined by Kocetkovs et al. [24] with minor adjustments. To release the bound forms of fatty acids, a solution of 10% (*w*/*v*) KOH dissolved in 80% MeOH (MeOH:H_2_O ratio 80:20 *v*/*v*) was utilized. This approach, under the dominance of MeOH, facilitated the hydrolysis and efficient release of fatty acids. In brief, triplicate samples (*n* = 3) of 3 ± 0.1 g of egg yolk obtained from 100 egg yolks and homogenized to form a uniform mass were weighed into 50 mL reagent bottles with screw caps. For hydrolysis, 30 mL of the prepared methanolic KOH solution was added to each egg yolk sample, and the mixture was incubated at 65 °C for 3 h in a water bath (Julabo^®^, Saalbach-Hinterglemm, Germany). Following hydrolysis, the release of fatty acids from the salt form was achieved by adjusting the pH from alkaline to acidic by adding 3.5 mL of HCl (6 M) until reaching pH 2.0. The extraction of the lipophilic fraction was carried out through liquid–liquid phase separation using *n*-hexane as the solvent. After cooling, the hydrolysates were transferred to falcon 50 mL conical centrifuge tubes, and 10 mL of *n*-hexane was added to each tube (Sarstedt AG & Co. KG, Nümbrecht, Germany), followed by vortex mixing for 1 min. The layers were separated by centrifugation in a Sigma, 2–16KC centrifuge (Osterode near Harz, Germany), and the top *n*-hexane layer was collected. This extraction process was repeated three times. The resulting lipophilic fraction (30 mL) was then evaporated using a Laborota 4002 rotary evaporator (Heidolph, Swabia, Germany) and re-dissolved in 5 mL of pyridine and filtered through a PTFE membrane filter with a pore size of 0.45 µm.

The TMPAH reagent was applied as a methylation agent of the functional groups to obtain volatile FAMEs derivatives. The methylation procedure was performed following the methodology described by Kocetkovs et al. [24].

The analysis of fatty acids (FAs) was carried out on a Clarus 600 system (PerkinElmer, Inc., Waltham, MA, USA) equipped with a single quadrupole Clarus 600 C mass-selective detector (Waltham, MA, USA). The optimized gas chromatography–mass spectrometry (GC-MS) conditions were implemented as per the protocol outlined by Kocetkovs et al. [24]. 

### 2.4. Sample Preparation and Analysis of Amino Acids by HPLC-ESI-TQ-MS/MS

The separation of AAs from the product matrix was performed according to the procedure described by Ruska et al. [15] with modifications, subjecting the sample to acid hydrolysis with 5 mL of 6 M HCl solution. Hydrolysis was performed in 22.0 mL glass Headspace chromatography vials (PerkinElmer, Inc., Waltham, MA, USA) with screw caps and silicone seals. The prepared sample (200 mg ± 0.1) in triplicates (*n* = 3) of an average egg white sample of 100 eggs (*n* = 100) was aged for 24 h in a drying cabinet “Pol-Eko Aparatura SP.J.”, (Wodzislava Slonska, Poland) at a temperature of 110 °C. During hydrolysis, the stabilizing reagent phenol, added directly to the sample at 0.02% (*w/w*), was used to delay the oxidation-reduction reaction of the compounds of interest. The volume of the hydrolysate obtained after hydrolysis was brought up to 10.0 mL with H_2_O and the pH of the medium was normalized to 6.5–6.8 using 2.64 mL of 25% ammonium hydroxide solution. Finally, the volume of the obtained hydrolysate was brought to 15.0 mL and subjected to intensive mixing for 1 min with a “ZX3” vortex mixer (Velp^®^ Scientifica, Usmate Velate, Italy). For the separation of fractions, the prepared hydrolysates were centrifuged for 10 min at 16,070× *g* and 19.0 ± 1 °C using a “Hermle Z 36 HK” centrifuge (Hermle Labortechnik, GmbH, Wehingen, Germany) and the upper organic layer was collected. Before LC-MS analysis, the collected layer was filtered through a 0.22 µm polytetrafluoroethylene (CROMAFIL^®^ Xtra H-PTFE) hydrophilized membrane filter (Macherey-Nagel GmbH & Co. KG, Dueren, Germany).

**Figure 1:**
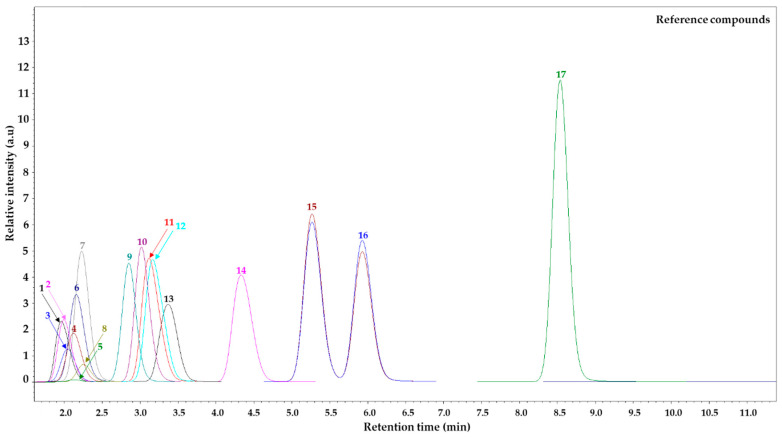
Selected ion chromatogram in MRM mode represents the profiles of 17 amino acids in a standard solution at the concentration of 2.5 μM L^−1^. *Note:* 1—cystine (Cys); 2—aspartic acid (Asp); 3—serine (Ser); 4—threonine (Thr); 5—glycine (Gly); 6—glutamic acid (Glu); 7—proline (Pro); 8—alanine (Ala); 9—histidine (His); 10—lysine (Lys); 11—valine (Val); 12—arginine (Arg); 13—methionine (Met); 14—tyrosine (Tyr); 15—isoleucine (Ile); 16—leucine (Leu); 17—phenylalanine (Phe).

AA analysis was accomplished using a “Shimadzu Nexera UC” series liquid chromatograph (LC) (Shimadzu Corporation, Tokyo, Japan) in tandem with a triple quadrupole mass selective detector (TQ-MS-8050, Shimadzu Corporation, Tokyo, Japan) equipped with electrospray ionization (ESI). Chromatographic separation of AAs was performed using a reversed-phase “Discovery^®^ HS F5-3” column (3.0 µm, 150 × 2.1 mm, Merck KGaA, Darmstadt, Germany) at a temperature of 40 °C and a flow rate of mobile phase 0.25 mL min^−1^. The sample injection of 3.0 μL was conducted automatically, and the loop was rinsed with 2-propanol. Mobile phase composition: acidified H_2_O (0.1% HCOOH *v/v*) (A) and acidified MeCN (0.1% HCOOH *v/v*) (B). The stepwise gradient elution program of mobile phase B during 15 min was programmed as follows: T_0_ min = 5.0%, T_2_ min = 5.0%, T_7.0_ min = 30.0%, T_11.0_ min = 60.0%, T_12.0_ min = 80.0%, T_12.1_ min = 5.0%. After each analysis, re-equilibration was completed for 3 min following the initial gradient conditions. A MeCN injection was included after each sample as a blank to avoid the carry-over effect. Data were acquired using “LabSolutions Insight LC-MS” version 3.7 SP3, which was also used for LC-MS control and data processing. This study used the positive electroionization mode, while data were collected in profile and centroid modes with a data storage threshold of 5000 absorption MS. Operating conditions of the mass-selective detector: detector voltage 1.98 kV, conversion dynode voltage 10.0 kV, interface voltage 4.0 kV, interface temperature 300 °C, desolvation line temperature 250 °C, heating block temperature 400 °C, spray gas—argon (Ar, purity 99.9%) with a flow rate of 3.0 L min^−1^, heating gas—carbon dioxide (CO^2^, purity 99.0%) with a flow rate of 10.0 L min^−1^, and drying gas—nitrogen (N^2^, separated from air using a nitrogen generator “Peak Scientific Instruments Ltd.” (Inchinan, Scotland, UK), purity 99.0%) with a flow rate of 10.0 L min^−1^. The AAs were observed in the programmed and optimized multiple reaction monitoring (MRM) mode, ensuring speed and accuracy and bypassing the process of derivatization [25]. Quantitative AAs analysis was executed at 15 °C by injecting 3.0 μL of a calibration solution in the concentration range from 0.075 to 2.5 μM L^−1^. The stock standard solution was prepared just before the analysis. Selective ion chromatogram (TIC) in MRM mode represents 17 AAs in Figure 1.

### 2.5. Physical Analysis of Eggs

The eggs were broken up, and the egg yolk and white were separated. The eggshell was washed and dried with absorbent paper. Eggshell thickness (mm) was measured at the equator with an accuracy of 0.001 mm by an electronic digital outside micrometer MT-21110X, (MRS Laboratory-Instruments, Harlow, UK) according to the methodology described by Sun et al. [26].

The color of the egg yolk was assessed using the ColorFlex^®^ EZ system (HunterLAB, Reston, VA, USA) based on the CIE Lab system with illuminant D65. Each sample underwent 30 measurements, with calibration against black and white tiles. The values for L* (100 = white, 0 = black), a* positive value (+) = red, a* negative value (−) = green, and b* positive value (+) = yellow, b* negative value (−) = blue were recorded to evaluate the color changes of eggs before and after the feeding trial. The total color difference ΔE was calculated (Equation (1)) [27], where a value of ΔE = 0 means no difference, while higher values indicate a greater color difference.

(1) $$\Delta E_{ab}=\sqrt{{{(L}_{2}^{*}-L_{1}^{*})}^{2}+{{(a}_{2}^{*}-a_{1}^{*})}^{2}+{{(b}_{2}^{*}-b_{1}^{*})}^{2}}$$

In the CIE Lab color space, the parameter chroma (C*) was determined using the Equation (2) [28]; when C* ≈ 0, the color is perceived as close to gray or neutral, while higher C* values indicate increased color saturation.

(2) $$C^{*}=\sqrt{{\left(a^{*}\right)}^{2}+{{(b}^{*})}^{2}}$$

### 2.6. Statistical Analysis

The obtained data were analyzed using a two-way mixed design analysis of variance (ANOVA) with the laying hens group (control and experimental) [29] for determination of effects of age and feed and their interaction (*p* < 0.05). Data are presented as the mean ± standard deviation of the mean (SD). Unless otherwise indicated, ANOVA test was used, which was followed by Student’s *t*-test. 

## 3. Results 

### 3.1. Laying Hens Performance and Quality Traits of Eggs

The present research demonstrated the significant impact of Lba on egg size (Table 3), especially on the ratio of medium (M) to extra-large (XL) eggs during stage 3. While the feeding stage did not influence the egg production performance (Table 3), the type of feed had a substantial influence. The two-way mixed-design ANOVA revealed no significant effect of stage and feed (*p* = 0.285), nor of stage (*p* = 0.675) or feed (*p* = 0.135) individually on eggshell thickness (Table 3). However, the effect of stage and feed was significant for egg yolk lightness (L*) during the feeding trial. Over the 12-week experiment, both groups showed a steady decrease in L* values, though yolks from hens receiving Lba supplementation were statistically lighter (*p* < 0.05) than those from the CON group. Regarding a* (redness) and b* (yellowness) values, yolks from the CON group were redder and yellower than those from the EXP group at all stages. The chroma (C*) value, indicating color saturation, also significantly differed (*p* < 0.05) based on the diet. Yolks from the EXP group had less saturated color than the CON group. This study observed notable differences in egg yolk color between stages and groups.

### 3.2. The Changes in Fatty Acid Profile and Quality Indices of Egg Yolk Lipids in Relation to Feeding Trial

The composition of FAs in lipids recovered from egg yolk is depicted in Table 4. In total, 16 FAs were identified and quantified, among which the dominance of oleic acid (C18:1n9c) from 45.84 to 47.57%, followed by palmitic acid (C16:0) from 24.48 to 28.18%, linoleic acid (C18:2n6c) from 9.32 to 13.47%, stearic acid (C18:0) from 7.93 to 9.30%, palmitoleic acid (C16:1n7c) from 2.66 to 4.08%, eicosatrienoic acid (C20:3n6c) from 0.01 to 2.70%, and vaccenic acid (C18:1n7t) from 0.71 to 1.30% was found. 

The most apparent reduction in the content of FAs was observed for vaccenic acid (C18:1n7t), corresponding to a 45.38% percentage loss. In contrast, an increase in the concentration of this MUFA representative was found in the experimental group (EXP) fed with the basic diet enriched with biotechnologically obtained Lba, corresponding to a 10.71% increase. Similar changes were observed for palmitoleic acid (C16:1n7c), corresponding to a 22.67% loss and a 10.41% increase for CON and EXP groups, respectively. A positive influence of biotechnologically obtained Lba was established for FAs such as palmitic (C16:0), stearic (C18:0), eicosanoic (C20:1n9c), and eicosadienoic (C20:2n6c) acids, corresponding to an increase of 1.88%, 5.56%, 100%, and 100%, respectively. In turn, no presence of the latter two FAs was revealed in the CON group. It was observed that the content of CLA, c9,t11-octadecadienoic acid (C18:2) in the CON and EXP groups was decreased by 1.49% and 9.64%, respectively. The concentration of CLA t10,c12-octadecadienoic acid (C18:2) in the CON group was higher by 3.64% and lower by 7.25% in the EXP group compared with the initial values.

### 3.3. The Changes in Amino Acid Profile and Quality Indices of Egg White in Relation to Feeding Trial

The analysis revealed the prevalence of Glu, Asp, Leu, Ser, Lys, and Val in egg white protein (Table 5). It is noted that the content of non-essential AAs, such as Glu and Ser, in the EXP group was statistically (*p* < 0.05) lower than those observed in the CON group at the first stage of the feeding trial. The content of these AAs in the EXP group remained substantially lower than in the CON group during the next 8 weeks of the dietary treatment. Estimating the content of essential AAs, such as Met and Val, it was observed that the EXP group contained the highest amount, while CON the lowest. Interestingly, the concentration of Met and Val after 12 weeks of dietary treatment for CON was not changed, though an increase of 3.81% and 2.11% was found in the EXP group. Considerable changes in AA content during dietary treatment were also observed for Asp, whose concentration in the EXP group increased significantly (*p* < 0.05) by 5.77% and 8.71% in stages 2 and 3 of the experiment, respectively. However, no statistically significant changes in the content of Asp were observed in the CON group (*p* > 0.05). The same trend of increase was noted for Leu, where its concentration was statistically (*p* < 0.05) higher in the EXP group after 12 weeks of the feeding trial. 

In stage 1, the CON group had the lowest sum of BCAAs and the highest value in the EXP group. Further dietary intervention for an additional four weeks (stage 2) and 8 weeks (stage 3) positively affected the content of BCAAs since the content in the EXP group was increased by 1.84% and 4.04%, respectively. In turn, no statistically significant (*p* > 0.05) changes in BCAAs were observed in the CON group after 8 or 12 weeks of the feeding trial. 

According to the results obtained (Table 5), already after four weeks of the feeding trial, the values of PER1, PER2, and PER3 for egg protein in the CON and EXP groups were statistically different (*p* < 0.05). The CON demonstrated that the values ranged from 2.90 to 3.00, while EXP ranged from 2.52 to 2.76. During the study, PER1, PER2, and PER3 values in the CON group did not statistically change and, at the end of the trial, remained the same as in stage 1 of the experiment. However, after 8 weeks of the feeding trial, the percentage increase in PER1, PER2, and PER3 values of the EXP group was statistically significant (*p* < 0.05), corresponding to a 6.15%, 6.56%, and 11.90% rise, respectively. The same trend of increase was observed at the end of the experiment, corresponding to a 12.19%, 8.49%, and 18.25% rise compared with the initial PER1, PER2, and PER3 values, respectively.

## 4. Discussion 

### 4.1. Laying Hens’ Performance and Quality Traits of Eggs

The egg production performance is the most critical commercial indicator of laying hen performance, which significantly affects the efficiency of the poultry industry as a whole. The eggs of M and L categories are two categories that are mostly sold because they correspond to consumer demands. The other egg categories are mainly used to obtain egg products. The present research demonstrates the significant impact of Lba on egg size, especially on the ratio of M to XL eggs obtained from hens in stage 3. According to Steenfeldt and Engberg [30], the maintenance of a healthy intestinal system in broilers is essential for achieving optimal growth, ultimately leading to an increase egg size. It is hypothesized that the positive effect of Lba on egg production performance was achieved due to its resistance to digestive enzymes and unique prebiotic activity that improved birds’ overall intestinal health [20]. The significantly higher egg production performance of hens in the EXP group provides economic benefits [31]. Thus, by using Lba in the diet of laying hens, it is possible to substantially increase the yield of eggs.

The results on eggshell thickness align with those reported by Sun et al. [26] indicating that it varied from 0.361 to 0.380 mm. The eggshell, along with its membranes, is of significant importance since it prevents eggs from mechanical damage and possible microbial invasion from the surrounding atmosphere. During their passage through the lower portion of the intestinal tract [32], the eggshell regulates water and gas exchange processes during egg development and storage [26,33]. Ray et al. [34] reported an increase in shell thickness and relative shell weight upon supplementing the diet of laying hens with probiotics. The results of the present study indicate a fairly obvious tendency for the eggshell thickening of eggs from EXP compared to the CON group. Moreover, the most evident contribution of dietary Lba was found after 8 and 12 weeks of the feeding trial. The increased thickness can be explained by more effective absorption and assimilation of calcium by laying hens due to a relatively lower gastric pH achieved by supplementing the diet with Lba and by promotion of fatty acids synthesis that, due to carboxyl groups (−COOH) at the end of backbone chains, are also acidic. This statement can be reinforced by an observation made by Guinotte et al. [35], indicating that the lower gastric pH, the better solubilization of calcium (in the form of calcium carbonate CaCO_3_) that can be achieved, and gastric acid secretion is a prerequisite for CaCO_3_, solubilization. Moreover, credible characterization of Lba as a strong calcium and other divalent cations chelator has been reported by Gobbetti and Gänzle [36], thus supporting the facts mentioned above. The higher assimilation of calcium by hens due to Lba may account for the initial pale color of egg yolks from the EXP group at the beginning of the dietary intervention. This observation aligns with findings by An et al. [37], suggesting that increased calcium levels in the diets of laying hens have an adverse effect on yolk color. As the dietary intervention progressed, a significant increase in the a* value of the EXP group and, to a lesser extent, the CON group was observed. This rise in a* value can be attributed to the ingestion of fat-soluble pigments, particularly carotenoids, from the diet of laying hens [38,39]. The darkening of the egg yolk was attributed to increased pigment accumulation, as indicated by a reduction in the L* value. The CON group showed a tendency towards decreased color saturation, with a noticeable decrease in the C* values. In contrast, the EXP group demonstrated no significant change in this parameter. The most notable color change (ΔE) was observed in the EXP group at stage 3 when compared to the initial value (stage 1), indicating a beneficial effect of Lba on the overall gastrointestinal environment of hens. This suggests that Lba promotes improved assimilation and stabilization of carotenoids from the diet by stimulating microflora and inhibiting lipid peroxidation [40,41].

### 4.2. The Changes in Fatty Acids and Quality Indices of Egg Yolk Lipids in Relation to Feeding Trial

The obtained results on FAs are consistent with those of Drabik et al. [42] and Kop-Bozbay et al. [43] indicating a nearly similar descending order of FA content recovered from the egg yolk of laying hens. The current study also revealed the absence of octadecatrienoic acid (ALA, C18:3n3c) and eicosapentaenoic acid (C20:5n3c) in egg yolk. The levels of these FAs in eggs are directly influenced by the dietary intake of laying hens as reported by Kralik et al. [44]. Specifically, studies have shown a notable increase in these acids when hens are provided with a diet high in flaxseeds [45,46]. The absence of omega-3-rich ingredients in the hens’ diet accounts for the lack of these fatty acids in the eggs.

Further analysis revealed that the control (CON) group receiving the basic diet during the 12-week experiment negatively affected the concentration of individual FAs in the egg yolk. Chamruspollert and Sell [47] reported similar fluctuations in CLA content in the egg yolk lipids, revealing that dietary origin has little or no effect on the FAs profile of egg yolk, including CLA. Overall, the content of FAs in egg yolk from laying hens was affected by the duration of the experiment and possibly the physiological state of laying hens caused by seasonality. However, without reference to relative fluctuations in the content of individual FAs, the higher values of SFAs were achieved by supplementing laying hens’ diets with Lba (EXP). Moreover, the dietary inclusion of biotechnologically produced Lba in the diet promoted the increase in the content of MUFA representatives, in particular, palmitoleic (C16:1n7c) acid and vaccenic (C18:1n7t) acids, the latter apart from being the dietary precursor of c9,t11 CLA reported to impart health benefits beyond those associated with CLA [48]. Based on the data obtained, no direct interconnection between the hens’ diet and the content of PUFA can be drawn in this study since the values are diametrically opposite at the same nutrient composition except for the EXP group, which contained an additional 2% of Lba in its composition. The observed cholesterol values are consistent with those reported by Zita et al. [49] for layers housed in the litter. The relatively higher percentage increase in cholesterol content in the CON group may be associated with the considerably lower production quantity of eggs laid by hens (Table 3). Given that the content of cholesterol in eggs is primarily associated with laying hens’ productivity and to a lesser extent with housing system, breed, age, and season, as reported by Zita et al. [49], the observed percentage reduction of cholesterol in this study can be somewhat attributed to the rate of eggs produced by layers from the EXP group that was much higher than that of CON group (Table 3). 

It was revealed that lipid indices, IA, IT, and HH in particular, are believed to be more accurate in predicting cardiovascular disease risk and providing insights into the metabolic health of individuals [50]. According to Chen and Liu [50], the lower the IA and IT values, the less risk of developing cardiovascular diseases caused by blood vessel clogging. The observed values of IA and IT are consistent with those reported by Vlaicu et al. [51] for lipids recovered from the egg yolk of laying hens fed different combined vegetable by-product diets rich in FAs and hydrophilic antioxidants. However, Omri et al. [52] reported substantially higher IA and IT values estimated for lipids recovered from the egg yolk of laying hens fed a linseed-rich diet alone or along with a tomato–red pepper mix. The differences in the values observed in the current study may be due to relatively lower saturated fatty acid (SFA) and higher MUFA and PUFA values, which are documented to provide cardioprotection and many other health-promoting benefits [53]. 

The observed HH indices are considerably higher than those reported by Paszczyk and Tońska [54], reinforcing the importance of eggs in the human diet. The decrease in HH values after 12 weeks of the dietary trial was supposed to be caused by the decrease in the PUFA ratio in the EXP group.

As demonstrated in earlier research, HPI can serve as a quality characteristic of dietary fat. Owing to the endower made by Chen et al. [55], HPI is now widely used to estimate the health-promoting properties of dairy-derived lipids. The higher the HPI value of the lipid, the more beneficial product to human health is [50]. Due to limitations in the scientific literature, no direct comparison of HPI values can be made. 

### 4.3. The Changes in Amino Acids and Quality Indices of Egg White in Relation to Feeding Trial

The detected AAs values are consistent with those reported by Carvalho et al. [9]. Unambiguously, it is challenging to say what caused the decrease in Glu content after 8 weeks of treatment. However, it is speculated that the conversion of this AA to nitrogen-containing compounds, such as glutamate and non-nitrogen-containing metabolites, such as glucose, may explain such a decline [56]. The observed values of essential AAs in the CON group align with those reported by Wang et al. [57]. It is worth noting that Martín-Venegas et al. [58] and Carvalho et al. [9] reported that the inclusion of sulfur-containing AAs in the diet of laying hens had negatively affected the amount of Leu and other branched-chain AAs (BCAAs) in eggs. 

BCAAs, including Leu, Ile, and Val, are documented to possess multiple beneficial effects on human organisms, including the therapeutic effect on hemoglobin A1C values in patients with marked peripheral (primarily muscle) insulin resistance [59], heart failure [60], and alleviation of skeletal muscle damage [61]. They must be dietary-obtained from food [62]. The observed values of BCAAs are consistent with those reported by Wang et al. [57] for hen dry egg white powder and nearly two-fold higher than that reported by Abd-Elaziz et al. [63]. 

Overall, supplementation of a basic hens’ diet with biotechnologically obtained Lba positively affected the content of individual AAs. The most apparent increase in the content of AAs, including BCAAs, was observed after 12 weeks of the feeding trial. The obtained results can be reinforced by data reported in previous work conducted by Ruska et al. [15] revealing the positive effect of Lba as a supplement to the diet of lactating cows on the overall AA profile in milk.

The predicted protein efficiency ratio (PER), described ten decades ago by Osborne et al. in 1919 [64], is a technique used to assess the quality of protein in foods by predicting the protein’s capability to sustain growth and maintenance in animals. The industry widely utilizes PER to estimate the nutritional value of raw materials and ready-food products. By using PER, manufacturers can determine the optimal protein sources to include in their products, ensuring high-quality protein and supporting optimal human growth and health. Given Friedman’s classification proposed almost two decades back, a PER < 1.5 is considered poor, from 1.5 to 2.0 is considered moderate, and >2.0 is considered superior [65]. As seen, the egg white derived from both groups, according to the quality criteria defined by the FAO/the World Health Organization (WHO), can be classified as highly digestible [66], and the PER values are relative to those documented for casein and whey protein [67]. The change in the essential to total AAs ratio (E/T) by enriching the diet of laying hens with biotechnologically obtained Lba affected the rise in PER values. Given the PER numbers, it is attainable to confirm the effectiveness of Lba in modifying the nutritional value of an already unique food product—eggs.

## 5. Conclusions

The availability of Lba in the whey fraction obtained through optimized fermentation designed by the group of LBTU utilizing *P*. *taetrolens* DSM 21104 was established chromatographically, corresponding to 11.3 ± 0.3 g L^−1^. A considerable yield of functional Lba made it feasible to enhance the laying hens’ (EXP) diet with the active component, which has been used to supplement the basic diet. The results of the study indicated an equally effective contribution of the Lba supplementation on laying hens’ productivity and egg quality traits during the early laying period. The diet supplementation with Lba significantly (*p* < 0.05) improved the egg production performance. Not significant, but still a relevant increase in the eggshell thickness has been observed in the experimental group, indicating a positive contribution of Lba to calcium assimilation by hens. The egg yolk color from the experimental group was significantly (*p* < 0.05) lighter than that of the control group, which may be considered a negative feature from consumers’ viewpoint. A substantial increase of MUFA, whereas a decrease of PUFA and cholesterol concentration (*p* < 0.05) was observed in eggs from the experimental group. Overall, Lba positively affected egg protein quality (*p* < 0.05), increasing BCAA content and PER values in the group and strengthening egg protein quality. 

## Figures and Tables

**Table 1:** Diet composition.

Item	Control Group (CON)	Experimental Group (EXP)
*Ingredients (%)*		
Wheat grain	40.73	40.44
Barley grain	19.64	19.50
Rapeseed	11.05	10.97
Soya bean meal	9.04	8.98
Sunflowers seeds	6.23	6.19
Vegetable oil	2.24	2.22
Limestone, different fractions	7.74	7.69
Premivit 3% Layer *	3.32	3.30
Lactobionic acid	0.00	0.72
*Calculated composition*		
Dry matter (%)	89.61	89.12
Crude protein (%)	15.92	15.83
Calcium (%)	2.93	2.91
Metabolizable energy (kcal kg^−1^)	2767	2751

*Note:* * Premivit 3% Layer (Vilomix Ltd., Mørke, Denmark) composition: calcium carbonate, mono calcium phosphorus, sodium bicarbonate, salt, MTB A+, additives: A, D_3_, E, B_1_, B_2_, B_6_, B_12_, K_3_, Ca-D-pantothen, niacinamide, biotin, choline chloride, folic acid, trace elements: ferrous sulphate monohydrate, iron, cupric sulphate, copper, manganous, manganese, zinc oxide, calcium iodate, sodium selenite, cobaltous, cobalt, amino acids: lysine, arginine, methionine. Chemical composition: ash—80%, calcium—19%, phosphorus—7%, sodium—5%, methionine—5%, lysine—3%.

**Table 2:** Physical–chemical characteristics of lactobionic acid used in the experiment as a supplement to the basic feed of laying hens.

Quality Trait	Composition of Lactobionic Acid Liquid Fraction	SD
Carbohydrates (%)	15.1 ^1^	0.7
Crude protein (%)	3.7	0.1
Crude fat (%)	0.06	0.01
pH	5.6	0.1
Lba (g L^−1^)	11.3	0.3
Dry matter (%)	22.5	1.2

*Note:* ^1^ Indicating the content of lactose solely in the whey fraction. Abbreviations: Lba = amount of lactobionic acid chromatographically quantified in the whey fraction after fermentation.

**Table 3:** Production results and quality traits of eggs obtained from hens fed differently at three laying stages.

Indices	CON Group			EXP Group			ANOVA *p*-Values		
							Main Effect		Interaction Effect
	*Stage 1*	*Stage 2*	*Stage 3*	*Stage 1*	*Stage 2*	*Stage 3*	*Stage*	*Group*	*Stage and Group*
S eggs (%)	12.30 ± 0.10 ^aB^	10.30 ± 0.20 ^bA^	3.90 ± 0.10 ^cB^	18.40 ± 0.20 ^aA^	10.10 ± 0.30 ^bA^	5.40 ± 0.10 ^cA^	2.47 × 10^−18^	2.02 × 10^−12^	3.63 × 10^−12^
M eggs (%)	75.80 ± 2.30 ^aA^	71.30 ± 1.20 ^bA^	59.50 ± 3.80 ^cB^	72.60 ± 2.40 ^aB^	70.50 ± 2.70 ^bA^	70.40 ± 2.30 ^bA^	0.001	0.076	0.001
L eggs (%)	11.60 ± 0.90 ^cA^	18.00 ± 1.10 ^bA^	36.50 ± 2.20 ^aB^	7.20 ± 0.90 ^cB^	15.20 ± 1.20 ^bB^	19.80 ± 1.10 ^aA^	4.50 × 10^−11^	2.18 × 10^−8^	1.44 × 10^−6^
XL eggs (%)	0.32 ± 0.04 ^aB^	0.28 ± 0.01 ^aB^	0.29 ± 0.02 ^aB^	0.90 ± 0.01 ^aA^	0.60 ± 0.02 ^bA^	0.60 ± 0.01 ^bA^	9.33 × 10^−9^	3.49 × 10^−14^	1.32 × 10^−7^
Egg production performance (%)	84.00 ± 2.00 ^aB^	85.00 ± 3.00 ^aB^	80.00 ± 2.40 ^bB^	91.20 ± 3.50 ^aA^	89.60 ± 3.30 ^abA^	88.90 ± 2.60 ^bA^	0.149	0.001	0.445
Eggshell thickness (mm)	0.35 ± 0.04 ^aA^	0.34 ± 0.03 ^aA^	0.36 ± 0.04 ^aA^	0.34 ±0.05 ^aA^	0.42 ± 0.04 ^aA^	0.42 ± 0.05 ^aA^	0.675	0.135	0.285
Egg yolk color, L*	47.30 ± 4.23 ^aB^	40.28 ± 5.39 ^bB^	39.40 ± 2.89 ^bB^	52.90 ± 3.45 ^aA^	50.68 ± 5.36 ^abA^	44.43 ± 2.47 ^bA^	0.016	0.004	0.486
Egg yolk color, a*	1.90 ± 0.12 ^bA^	3.29 ± 0.08 ^aA^	4.20 ± 0.41 ^aA^	1.20 ± 0.04 ^cA^	2.20 ± 0.01 ^bB^	3.24 ± 0.02 ^aA^	0.30 × 10^−10^	1.50 × 10^−7^	0.216
Egg yolk color, b*	30.34 ± 1.23 ^aA^	27.20 ± 1.78 ^bA^	27.80 ± 3.45 ^bA^	25.80 ± 4.45 ^aB^	25.40 ± 3.18 ^aB^	25.90 ± 4.12 ^aB^	0.635	0.098	0.713
C*	30.40 ± 2.74 ^aA^	27.39 ± 2.45 ^aA^	28.12 ± 1.98 ^aA^	25.83 ± 2.68 ^aB^	25.50 ± 1.88 ^aB^	26.10 ± 2.67 ^aB^	0.084	0.003	0.949
ΔE_stage_	–	7.03 ±1.14	7.90 ± 0.97	–	2.47 ± 1.04	8.71 ± 0.99	NA	NA	NA
ΔE_group_	–	–	–	7.24 ± 1.01	10.61 ± 0.84	5.46 ± 1.48	NA	NA	NA

*Note*. Egg production performance and egg size were evaluated daily for all hens, with approximately 60,000 eggs assessed per group per experiment. Values are means ± SD of triplicates (*n* = 3) with 30 eggs (*n* = 30) used for color assessment. Different lowercase (^a,b,c^) and uppercase (^A,B^) superscripts in the same row indicate significant differences (Student’s *t*-test; *p* < 0.05) between laying stage and study groups, respectively. Abbreviations: S, M, L, XL indicate egg size, where: S—small egg up to 52 g; M—middle egg up to 53–62 g; L—large egg up to 63–72 g; XL—extra-large egg more than 72 g; L*—lightness (100 = white, 0 = black); a*—positive value (+) = red, negative value (−)—green; b*—positive value (+) = yellow, negative value (−)—blue; CON—control group received the basic diet; ΔE_stage_—the total color differences between stage of the same groups; ΔE_group_—the total color differences between groups at different stages; C*—chroma, EXP—experimental group received lactobionic acid as a supplement to the basic diet; NA—not applicable.

**Table 4:** The profile of fatty acids, cholesterol content in egg yolk lipids, and comparison of quality indices between the groups and laying stages.

Indices	Abbreviation	Study Group						ANOVA *p*-Values	
		CON			EXP			Main Effect	
		*Stage 1*	*Stage 2*	*Stage 3*	*Stage 1*	*Stage 2*	*Stage 3*	*Stage*	*Group*
*Fatty acid (% DW)*									
Hexadecanoic acid	C16:0	24.48 ^bB^	25.98 ^aB^	23.71 ^cB^	27.66 ^bA^	27.66 ^bA^	28.18 ^aA^	<0.05	<0.05
Hexadecenoic acid	C16:1n7c	3.44 ^bA^	4.08 ^aA^	2.66 ^cA^	2.69 ^bB^	2.96 ^abB^	2.97 ^aA^	<0.05	<0.05
Heptadecanoic acid	C17:0	BLQ	0.01 ^bA^	BLQ	BLQ	BLQ	BLQ	-	-
Octadecanoic acid	C18:0	7.93 ^bB^	8.73 ^aA^	7.78 ^bB^	8.81 ^bA^	8.81 ^bA^	9.30 ^aA^	<0.05	<0.05
Octadecenoic acid	C18:1n9c	45.94 ^bB^	45.84 ^bB^	46.76 ^aB^	47.16 ^aA^	47.57 ^aA^	47.35 ^aA^	<0.05	<0.05
Octadecenoic acid	C18:1n7t	1.30 ^aA^	0.81 ^bB^	0.71 ^bB^	0.84 ^cB^	1.09 ^aA^	0.93 ^bA^	<0.05	<0.05
Octadecadienoic acid	C18:2n6c	11.15 ^bA^	9.32 ^cA^	13.47 ^aA^	10.58 ^aB^	9.64 ^bA^	9.11 ^cB^	<0.05	<0.05
Octadecatrienoic acid	C18:3n6c	1.00 ^bA^	0.86 ^cA^	1.43 ^aA^	0.39 ^aB^	0.28 ^aB^	0.28 ^aB^	<0.05	<0.05
CLA, c9,t11-Octadecadienoic acid	C18:2	0.67 ^aB^	0.61 ^aA^	0.66 ^aA^	0.83 ^aA^	0.79 ^aA^	0.75 ^aA^	>0.05	<0.05
CLA, t10,c12 Octadecadienoic acid	C18:2	0.55 ^aA^	0.52 ^aA^	0.57 ^aA^	0.69 ^aA^	0.65 ^aA^	0.64 ^aA^	>0.05	>0.05
Eicosenoic acid	C20:1n9c	n.d.	n.d.	n.d.	n.d.	0.19 ^a^	0.16 ^a^	>0.05	-
Heneicosanoic acid	C21:0	n.d.	n.d.	n.d.	0.07	n.d.	n.d.	-	-
Eicosadienoic acid	C20:2n6c	n.d.	n.d.	n.d.	n.d.	0.05 ^a^	0.05 ^a^	>0.05	-
Eicosatrienoic acid	C20:3n6c	2.70 ^aA^	2.53 ^bA^	2.28 ^cA^	0.01 ^aB^	0.01 ^aB^	0.01 ^aB^	<0.05	<0.05
Eicosatetraenoic acid	C20:4n6c	n.d.	n.d.	n.d.	0.24 ^aA^	0.25 ^aA^	0.24 ^aA^	>0.05	-
Docosahexaenoic acid	C22:6n3c	0.84 ^aA^	0.71 ^abA^	0.67 ^bA^	0.03 ^aB^	0.04 ^aB^	BLQ	<0.05	<0.05
∑_SFAs_		32.41 ^bB^	34.71 ^aB^	31.49 ^cB^	36.54 ^bA^	36.47 ^bA^	37.48 ^aA^	<0.05	<0.05
∑_MUFAs_		50.68 ^aA^	50.73 ^aB^	49.42 ^bB^	50.68 ^bA^	51.81 ^aA^	51.42 ^aA^	<0.05	<0.05
∑_PUFAs_		16.90 ^bA^	14.56 ^cA^	19.09 ^aA^	12.78 ^aB^	11.72 ^bB^	11.10 ^cB^	<0.05	<0.05
∑_n-3_		0.84 ^aA^	0.71 ^aA^	0.67 ^a^	0.03 ^bB^	0.04 ^bB^	n.d.	>0.05	<0.05
∑_n-6_		16.07 ^bA^	13.84 ^cA^	18.41 ^aA^	12.74 ^aB^	11.67 ^aB^	11.08 ^aB^	<0.05	<0.05
CLA		1.22 ^aA^	1.14 ^aA^	1.23 ^aA^	1.52 ^aA^	1.44 ^aA^	1.39 ^aA^	>0.05	>0.05
PUFA/SFA		0.52 ^bA^	0.42 ^cA^	0.61 ^aA^	0.35 ^aB^	0.32 ^aB^	0.30 ^aB^	<0.05	<0.05
Cholesterol (mg 100^−1^ g DW)		1170.00 ± 94.54 ^bB^	1456.00 ± 13.59 ^aB^	1146.73 ± 60.26 ^cB^	1590.34 ± 95.43 ^aA^	1538.05 ± 15.71 ^bA^	1445.14 ± 49.31 ^cA^	<0.05	<0.05
IA		0.05 ^aA^	0.06 ^aA^	0.04 ^aA^	0.04 ^aA^	0.05 ^aA^	0.05 ^aA^	>0.05	>0.05
IT		0.46 ^aA^	0.44 ^aA^	0.50 ^aA^	0.43 ^aA^	0.41 ^aA^	0.40 ^aA^	>0.05	>0.05
HH		4.91 ^bA^	3.57 ^cA^	7.18 ^aA^	4.75 ^aA^	3.96 ^bA^	3.74 ^bB^	<0.05	<0.05
HPI		19.65 ^bB^	16.00 ^cB^	25.76 ^aA^	23.59 ^aA^	21.46 ^bA^	21.05 ^bB^	<0.05	<0.05

*Note*: Values are means ± SD of triplicates (*n* = 3) of an average egg yolk sample of 100 eggs (*n* = 100). Different lowercase (^a,b,c^) and uppercase (^A,B^) superscripts in the same row indicate significant differences (Student’s *t*-test; *p* < 0.05) between laying stage and study groups, respectively. Abbreviations: CON—control group received the basic diet; EXP—experimental group received lactobionic acid as a supplement to the basic diet; SFA—saturated fatty acids; MUFA—monounsaturated fatty acids; PUFA—polyunsaturated fatty acids; CLA—conjugated linoleic acid; IA—index of atherogenicity; IT—index of thrombogenicity; HH—ratio of hypocholesterolemic to hypercholesterolemic levels; HPI—health-promoting index; BLQ—below limit of quantification; n.d.—not detected; DW—the amount of a respective fatty acid is expressed as the relative amount to total fatty acids (percentage of total fatty acid) on a dry weight basis.

**Table 5:** The profile of amino acids in egg white and comparison of quality indices between the groups and laying stages.

Indices	Study Group						ANOVA *p*-Values	
	CON			EXP			Main Effect	
	*Stage 1*	*Stage 2*	*Stage 3*	*Stage 1*	*Stage 2*	*Stage 3*	*Stage*	*Group*
*Amino acids (* *g 100 g^−1^ DW)*								
Alanine (Ala)	5.82 ± 0.10 ^aB^	5.89 ± 0.21 ^aB^	5.82 ± 0.20 ^aB^	6.54 ± 0.11 ^aA^	6.46 ± 0.33 ^aA^	6.63 ± 0.00 ^aA^	<0.05	<0.05
Arginine (Arg)	5.58 ± 0.12 ^aA^	5.51 ± 0.11 ^aA^	5.57 ± 0.12 ^aA^	5.62 ± 0.11 ^aA^	5.63 ± 0.44 ^aA^	5.84 ± 0.00 ^aA^	>0.05	>0.05
Aspartic acid (Asp)	10.53 ± 0.10 ^aA^	10.40 ± 0.11 ^aB^	10.52 ± 0.11 ^aB^	10.74 ± 0.11 ^bA^	11.36 ± 0.76 ^aA^	11.67 ± 0.20 ^aA^	<0.05	<0.05
Cysteine (Cys)	2.60 ± 0.13 ^aA^	2.63 ± 0.15 ^aB^	2.60 ± 0.11 ^aB^	2.97 ± 0.11 ^aA^	3.02 ± 0.22 ^aA^	3.07 ± 0.10 ^aA^	>0.05	<0.05
Glycine (Gly)	3.47 ± 0.30 ^aA^	3.51 ± 0.23 ^aA^	3.59 ± 0.73 ^aA^	3.78 ± 0.85 ^aA^	3.54 ± 0.44 ^aA^	2.77 ± 0.20 ^bB^	>0.05	<0.05
Glutamine (Glu)	13.51 ± 0.31 ^aA^	13.53 ± 0.12 ^aA^	13.49 ± 0.29 ^aA^	7.98 ± 0.21 ^aB^	7.92 ± 0.44 ^aB^	8.31 ± 0.10 ^aB^	>0.05	<0.05
Proline (Pro)	3.97 ± 0.12 ^aA^	3.88 ± 0.08 ^aA^	3.96 ± 0.16 ^aA^	3.89 ± 0.11 ^aA^	3.75 ± 0.21 ^aA^	3.96 ± 0.00 ^aA^	>0.05	>0.05
Serine (Ser)	6.69 ± 0.10 ^aA^	6.64 ± 0.15 ^aA^	6.68 ± 0.15 ^aA^	6.03 ± 0.85 ^aB^	6.04 ± 0.23 ^aB^	3.96 ± 0.71 ^bB^	>0.05	<0.05
Histidine (His)	2.35 ± 0.12 ^aA^	2.38 ± 0.14 ^aA^	2.35 ± 0.11 ^aA^	2.25 ± 0.11 ^aA^	2.29 ± 0.11 ^aA^	2.37 ± 0.00 ^aA^	>0.05	>0.05
Isoleucine (Ile)	5.70 ± 0.21 ^aB^	5.64 ± 0.15 ^aB^	5.69 ± 0.15 ^aB^	6.13 ± 0.21 ^aA^	6.35 ± 0.22 ^aA^	6.33 ± 0.20 ^aA^	>0.05	<0.05
Leucine (Leu)	8.55 ± 0.31 ^aA^	8.65 ± 0.21 ^aA^	8.54 ± 0.18 ^aA^	8.18 ± 0.21 ^bA^	8.54 ± 0.22 ^abA^	8.70 ± 0.20 ^aA^	<0.05	>0.05
Lysine (Lys)	6.82 ± 0.12 ^aB^	6.89 ± 0.13 ^aB^	6.81 ± 0.27 ^aB^	7.57 ± 0.11 ^aA^	7.50 ± 0.44 ^aA^	7.72 ± 0.00 ^aA^	>0.05	<0.05
Tyrosine (Tyr)	4.09 ± 0.12 ^aB^	4.01 ± 0.12 ^aA^	4.08 ± 0.10 ^aB^	4.60 ± 0.11 ^aA^	4.48 ± 0.22 ^aA^	4.65 ± 0.00 ^aA^	>0.05	<0.05
Threonine (Thr)	4.46 ± 0.10 ^aB^	4.39 ± 0.10 ^aB^	4.46 ± 0.18 ^aB^	5.11 ± 0.00 ^aA^	5.10 ± 0.33 ^aA^	5.24 ± 0.00 ^aA^	>0.05	<0.05
Valine (Val)	6.44 ± 0.25 ^aB^	6.52 ± 0.43 ^aB^	6.44 ± 0.31 ^aB^	7.36 ± 0.11 ^aA^	7.19 ± 0.44 ^aA^	7.52 ± 0.00 ^aA^	>0.05	<0.05
Methionine (Met)	3.47 ± 0.09 ^aB^	3.51 ± 0.18 ^aA^	3.47 ± 0.18 ^aB^	4.19 ± 0.00 ^aA^	3.96 ± 0.22 ^aA^	4.35 ± 0.00 ^aA^	>0.05	<0.05
Phenylalanine (Phe)	5.95 ± 0.14 ^aB^	6.02 ± 0.14 ^aB^	5.94 ± 0.19 ^aB^	7.06 ± 0.21 ^bA^	6.77 ± 0.22 ^cA^	7.92 ± 0.00 ^aA^	<0.05	<0.05
∑_Essential AAs_	49.32 ± 1.34 ^aB^	49.50 ± 1.59 ^aB^	49.26 ± 1.69 ^aB^	53.48 ± 1.07 ^bA^	53.33 ± 2.64 ^bA^	55.00 ± 0.40 ^aA^	<0.05	<0.05
∑_Branched-chain AAs_	20.69 ± 0.77 ^aB^	20.80 ± 0.79 ^aB^	20.67 ± 0.64 ^aB^	21.68 ± 0.53 ^bA^	22.08 ± 0.87 ^ab^	22.55 ± 0.41 ^aA^	<0.05	<0.05
*Amino acid quality indices*								
PER_1_	2.90 ^aA^	2.95 ^aA^	2.90 ^aB^	2.76 ^bA^	2.93 ^abA^	3.10 ^aA^	<0.05	<0.05
PER_2_	2.97 ^aA^	3.02 ^aA^	2.96 ^aA^	2.74 ^bA^	2.92 ^abA^	2.98 ^aA^	<0.05	>0.05
PER_3_	3.00 ^aA^	3.17 ^aA^	2.99 ^aA^	2.52 ^bB^	2.82 ^abB^	2.98 ^aA^	<0.05	<0.05
E/T (%)	0.48 ^aA^	0.48 ^aA^	0.48 ^aA^	0.52 ^aA^	0.52 ^aA^	0.54 ^aA^	>0.05	>0.05

*Note:* Values are means ± SD of triplicates (*n* = 3) of an average egg white sample of 100 eggs (*n* = 100). Different lowercase (^a,b,c^) and uppercase (^A,B^) superscripts in the same row indicate significant differences (Student’s *t*-test; *p* < 0.05) between laying stage and study groups, respectively. Abbreviations: CON—control group received the basic diet; EXP—experimental group received lactobionic acid as a supplement to the basic diet; AA—amino acid; PER—protein efficiency ratio; E/T—the ratio of essential amino acids (E) to the total amino acids (T); DW—the amount of a respective amino acid is expressed as g 100 g^−1^ on a dry weight basis. Branched-chain amino acids are a sum of essential amino acids, including leucine, isoleucine, and valine.

## Data Availability

Data are available from authors upon reasonable request.
